# Advanced Characterization of Environmental Pollutant Metabolism in Human Skin

**DOI:** 10.3390/jox15050163

**Published:** 2025-10-11

**Authors:** Rafael Reis, Martine Zanini, Guillaume Lereaux, Ariane Dimitrov, Samia Boudah

**Affiliations:** 1 Department of Environmental Toxicology, Eawag, Swiss Federal Institute of Aquatic Science and Technology, 8600 Dübendorf, Switzerland; rafael.teixeiradosreis@eawag.ch; 2 Exposome Discovery Domain, L’Oreal Research & Innovation, 93600 Aulnay sous Bois, France; martine.zanini@loreal.com (M.Z.); ariane.dimitrov@loreal.com (A.D.); 3 ADME Laboratory, L’Oréal Research & Innovation, 93600 Aulnay sous Bois, France; guillaume.lereaux@loreal.com; 4 Analytical Chemistry Department, L’Oréal Research & Innovation, 93600 Aulnay sous Bois, France

**Keywords:** skin metabolism, PAH, LC-HRMS, isotope labeling, data mining

## Abstract

Ultrafine particles (UFPs) containing polycyclic aromatic hydrocarbons (PAHs) benzo[a]pyrene (BaP), are linked to pollution-induced health concerns, with skin being highly susceptible to contamination. Understanding the metabolic fate of these environmental pollutants in the skin is crucial. Moreover, traditional in vitro models often lack metabolic competency, while animal testing raises ethical concerns. This study introduces a novel approach combining stable isotope labeling (SIL) and liquid chromatography–high-resolution mass spectrometry (LC-HRMS) to investigate BaP metabolism. The physiologically relevant 3D reconstructed human epidermis (RHE) model was used. RHE models were exposed to BaP and deuterium-labeled BaP (BaP-d12). These analyses, followed by data analysis incorporating stable isotope filtering, revealed the presence of five distinct BaP phase I metabolites, including mono-hydroxylated, dihydroxylated, and quinone derivatives. This study demonstrates the power of coupling stable isotope labeling with LC-HRMS for the comprehensive characterization of BaP metabolic pathways in human skin. The identification of specific metabolites enhances our understanding of BaP detoxification mechanisms and their potential adverse effects. This analytical approach holds promise for investigating the metabolic fate of various other environmental pollutants.

## 1. Introduction

The skin is continuously exposed to various environmental stressors that differ in both nature and intensity [1]. These include physical factors such as ultraviolet (UV) radiation and temperature changes, as well as chemical exposures like irritants, allergens, and pollutants [1,2,3]. Among these stressors, air pollution stands out due to its complex composition and pervasive impact on skin health [4].

Air pollution consists of numerous chemical substances, with fine and ultrafine particles ranking among the most harmful. These particles, often generated by the combustion of fossil fuels such as wood and coal, serve as carriers for toxic compounds including heavy metals and polycyclic aromatic hydrocarbons (PAHs). Within the broad class of PAHs, benzo[a]pyrene (BaP) is one of the most extensively studied due to its environmental prevalence and biological effects [5]. These contaminants access the skin through systemic exposure or direct dermal exposure then subsequently exacerbate oxidative stress and inflammation, posing a significant threat to cutaneous health [6,7]. PAHs have also been detected systemically, with reported blood concentrations in the nanomolar range [8]. Additionally, in occupational settings, such as among asphalt workers, dermal exposure studies have reported PAH concentrations on the skin in the low ng/cm^2^ range [9].

Clinical studies have reported a higher incidence of pigmentary disorders, acne, severe seborrheic conditions, and disruptions in the skin microbiome among individuals exposed to environmental pollutants [7,10,11]. These previous findings are corroborated by in vitro experiments using keratinocytes and reconstructed epidermal models (both 2D and 3D), which show that exposure to PAHs disrupts cellular and mitochondrial redox homeostasis [12,13]. One prevailing hypothesis is that the metabolic activation of PAHs enhances their reactivity and ability to bind DNA, thereby increasing their toxic potential [14]. Although BaP is not directly toxic in its original form, it can significantly increase photoreactivity and phototoxicity when skin cells are exposed to UVA1 radiation (350–400 nm) [13,15]. To understand these effects further, an investigation into BaP xenometabolism is crucial.

To eliminate such lipophilic xenobiotic molecules, the body relies on multi-step enzymatic processes that transform them into more hydrophilic, excretable compounds. These metabolic detoxification mechanisms are active in several organs susceptible to xenobiotic accumulation—such as the liver, kidneys, and lungs—and are also well-developed in the skin, reflecting its critical role as a barrier and first line of defense [16,17,18]. However, certain metabolic pathways can lead to the formation of biologically reactive metabolites. Specifically, BaP undergoes metabolic activation via xenobiotic-metabolizing enzymes, particularly cytochrome P450 monooxygenases (CYP) and microsomal epoxide hydrolase (mEH), resulting in highly mutagenic and carcinogenic intermediates [19].

Given the potential for BaP and other xenobiotics to form harmful metabolites during metabolism, accurately identifying and characterizing skin metabolites is essential for understanding their toxicological profiles and potential risks to skin health. This need has driven the development and application of advanced analytical techniques capable of detecting a wide range of known and unknown transformation products (TPs).

The development of high-resolution mass spectrometry (HRMS) instruments—such as Orbitrap MS and quadrupole time-of-flight MS (QTOF-MS)—has significantly advanced suspect and non-targeted screening approaches for the detection of unknown metabolites. These instruments deliver high full-scan sensitivity (ranging from femtogram to picogram levels) combined with high mass resolution (up to 1,000,000 full width at half-maximum, FWHM) and mass accuracy (typically <5 parts per million, ppm). These features enable precise determination of molecular formulas based on exact mass and isotopic pattern analysis. In addition, HRMS allows for retrospective data analysis, enabling researchers to screen for newly suspected metabolites without the need for reinjection of samples [20]. However, the complexity and volume of data generated by these instruments pose a major challenge. Efficient data processing and interpretation require comprehensive, systematic data-mining strategies to extract relevant signals, improve analytical throughput, and enhance the identification of both known and unknown compounds.

Recent advances in data-mining strategies have significantly enhanced TP identification, including approaches such as suspect screening based on in silico prediction, stable isotope labeling, mass defect filtering, and chemometrics, among others [21,22]. Among these, stable isotope labeling (SIL) stands out as a particularly powerful technique for distinguishing ions of interest within complex mixtures. For instance, Takahashi et al. used this approach to identify 16 metabolites of 2,4-dichlorophenoxyacetic acid [23]. SIL enables the identification of ion pairs that differ by a specific mass-to-charge (*m*/*z*) value (Δ*m*/*z*), corresponding to the labeled and unlabeled forms of a compound [24].

This strategy has been successfully applied in both metabolomics and environmental research, including the identification of pesticide TPs [25,26,27]. However, to the best of our knowledge, no studies have employed SIL for the investigation of BaP metabolites or in the cosmetic field (skin metabolism). In this context, we took advantage of our previously published study which BaP accumulation in skin was assessed by absolute quantification [12]. The present study aims to complement this work by exploring the xenometabolism of BaP in reconstructed 3D skin models using stable SIL combined with HRMS. By applying SIL, we seek to enhance the identification of BaP-derived TPs, including those previously undetected through conventional approaches. In order to unveil the interplay between the skin and the pollutant, our primary objective is twofold: (i) to optimize a SIL-HRMS workflow tailored for skin xenobiotic metabolism studies and (ii) to comprehensively uncover then annotate and identify both known and novel BaP metabolites generated in skin models.

## 2. Materials and Methods

### 2.1. Materials and Chemicals

Benzo[a]pyrene (BaP) (CAS 50-32-8) and Benzo[a]pyrene-d12 (BaP-d12) (CAS 63466-71-7) at 98% purity were purchased from Dr. Ehrenstorfer via LGC standards (Augsburg, Germany) and Cambridge Isotope Laboratories (Tewksbury, MA, USA), respectively.

All solvents and reagents used in this study were obtained from Sigma-Aldrich (St. Louis, MO, USA). HPLC–MS-grade acetonitrile and methanol were acquired from Merck (Lowe, NJ, USA), while laboratory-grade ethanol was acquired from Merck Millipore (Darmstadt, Germany). HPLC-grade water was prepared using a Milli-Q Integral 15 system (Merck Millipore, Burlington, MA, USA).

### 2.2. Chemical Treatment of RHE Tissues

Reconstructed human epidermis (RHE) D10 tissues (Episkin^®^, Lyon, France), cultured on polycarbonate membranes, were maintained in the recommended SkinEthic growth medium. Following 10 days of differentiation, the tissues were exposed for three consecutive days (from day 11 to day 14) to culture medium containing either 0.1% DMSO (vehicle control) or 2.5 µg/mL of BaP or BaP-d12 in order to mimic systemic exposure. After each 24-h exposure period, the tissues were transferred to fresh culture plates with refreshed medium. The separation of the epidermis from the polycarbonate membrane was performed on day 14 and acetonitrile (ACN) was used to extract the compounds of interest separately from the epidermis. For each experiment, tissues derived from a single donor were used to perform two parallel exposure conditions—BaP and BaP-d12—each in three biological replicates.

**Figure 1:**
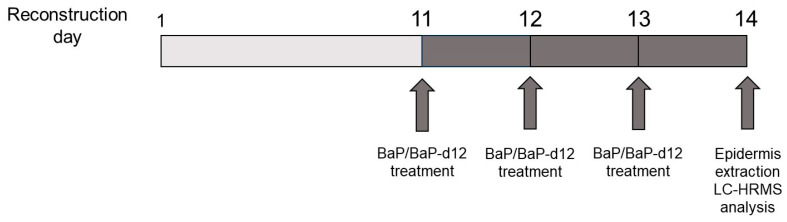
Experimental timeline for repeated BaP/BaP-d12 exposure of reconstructed human epidermis (RHE).

As previously reported [12,13], the effective concentration of PAHs reaching the cells is lower than the nominal concentration, due to the strong affinity of these compounds for plastic surfaces, resulting in significant losses during preparation. Figure 1 illustrates the experimental design used for these in vitro exposure assays.

### 2.3. Chemical Analysis

Chemical analysis was performed using an Ultimate 3000 ultra-high-performance liquid chromatography (UHPLC) system coupled to a photodiode array (PDA) detector and an Orbitrap Fusion high-resolution mass spectrometer (HRMS), equipped with atmospheric pressure chemical ionization (APCI) (Thermo Fisher Scientific, Waltham, MA USA). Chromatographic separation was performed on a Pinnacle II PAH analytical column (150 mm × 3.2 mm; 4 µm), coupled to a Pinnacle II PAH pre-column (10 mm × 4.0 mm), under isocratic conditions with a mobile phase of acetonitrile/water (95:5, *v*/*v*) at a flow rate of 1.2 mL/min. A 30 µL injection volume was used. Samples were ionized via APCI in positive mode. Spray current in positive ion discharge was set at 10 µA. Sheath, auxiliary and sweep gas parameters were set to 30, 10 and 5 arbitrary units. Ion transfer temperature was at 325°. Data were acquired in full-scan mode over an *m*/*z* range of 50–500, with a resolution of 120,000 FWHM at *m*/*z* 200. The instrument was calibrated before use following the manufacturer’s recommendations.

### 2.4. Data Analysis

The raw mass spectrometry (MS) data were processed using Compound Discoverer^®^ 3.1 (Thermo Fisher Scientific), with a customized workflow adapted for peak detection, alignment, ion pairing, and annotation. Accordingly, the LC-HRMS profiles of BaP and BaP-d12 samples were analyzed using untargeted metabolite screening. Signal extraction was performed with the following parameters: a minimum intensity threshold of 1000, a mass tolerance of 10 ppm, and consideration of the following adducts: [M + H]^+^, [M − H]^−^, [M + 2H]^2+^, [M − 2H]^2−^, [2M + H]^+^, [2M − H]^−^, [M + H − H_2_O]^+^, and [M − H − H_2_O]^−^. Peaks were then aligned across all the samples and gaps were filled. Hydrogen and deuterium were selected for the ion pairing highlight. Finally, for annotation, the maximum elemental composition was set to C_90_H_190_^2^H_20_N_10_O_15_ within a tolerance of 10 ppm, while all other parameters were kept at their default settings.

This automated workflow transforms raw LC-HRMS files into a structured output, producing a final Excel .xls table summarizing detected features (*m*/*z* and retention time) and their corresponding metadata such as ion pairs.

### 2.5. Workflow Validation

As qualitative results must be underpinned by a robust validation framework, we have applied a comprehensive validation strategy for our untargeted approach, explicitly following the three essential steps described by Evans et al. (*Metabolomics* 2020, 16(10): 113) [28] and other best practices for untargeted metabolomics:

1. Quality Assurance (QA) Practices:
  - The analytical setup and workflows strictly adhere to Standard Operating Procedures (SOPs).
  - The LC-HRMS instrument used was systematically calibrated, tuned, and maintained according to the manufacturer’s specifications and our internal QA protocols prior to each analytical sequence. This includes regular checks of mass accuracy and signal intensity, ensuring the instrument is operating optimally for untargeted metabolite detection.
2. Quality Control (QC) Sample Implementation:
  - Replicate Samples: Experimental samples were run in triplicates. This allowed us to evaluate the consistency of metabolite detection across technical replicates, ensuring that detected features were robustly observed.
  - Internal Standards (IS): BaP and BaP 12D were qualitatively considered as internal standards. They served as end-to-end process controls, allowing us to monitor and assess the consistency of extraction efficiency, chromatographic alignment, ion pairing, and overall data treatment performance throughout the entire analytical workflow.
  - Blank Samples: Process blanks were strategically interspersed within the analytical batch. These blanks were instrumental in identifying potential background noise, reagent contaminants, and carryover effects. Features detected in process blanks that exceeded a predefined signal-to-noise ratio were filtered out to enhance the specificity of our xenometabolite detection.
3. Post-Analysis Quality Management:
  - Data Processing and Filtering: We utilized the Compound Discoverer algorithm, which integrates an XCMS package, to process the raw data. While such algorithms are extensively validated for peak detection and alignment [29], we implemented additional manual review steps, as recommended by Evans et al., to verify peak detection, chromatographic alignment, and feature integration for critical xenometabolites.
  - Quality of Identification: For the characterization of xenometabolites, we followed Metabolomics Standards Initiative (MSI) recommendations. This involves matching exact mass, retention time, and MS/MS fragmentation patterns against authentic standards or spectral libraries when possible. The highlighted metabolites were all in the 3rd level of confidence (exact mass and formula match).

## 3. Results

### 3.1. Stable Isotope Labeling Approach

Previous studies have demonstrated that BaP exposure alters the metabolic profile of skin cells, particularly through the activation of xenobiotic metabolism pathways [30]. To distinguish BaP-derived metabolites from endogenous ones and improve analytical specificity, a stable isotope-labeled form of BaP (BaP-d12) was used. Given the comparable physicochemical properties of native BaP and its stable isotope-labeled analog (BaP-d12), it is expected that both compounds elicit similar biological effects and undergo the same metabolic transformations, leading to the formation of identical xenometabolites.

**Figure 2:**
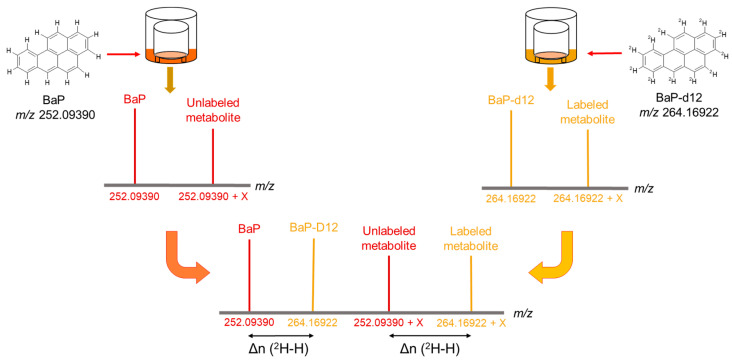
Schematic representation of the application of isotopic labeling to metabolite identification. Cells from the same donor are treated in parallel with BaP and BaP-d12. X: functional group indicating a metabolic reaction; Δn (^2^H–H): deuterium–hydrogen exchange number.

To minimize intrinsic biological variability between donors, cells derived from the same donor were treated in parallel with either BaP or BaP-d12, allowing for direct comparison (Figure 2). The incorporation of this labeled compound allows for precise tracking of metabolic transformations of BaP through characteristic isotopic patterns and mass shifts. These xenometabolites include both phase I BaP metabolites generated via functionalization of biotransformation pathways (e.g., cytochrome P450-mediated oxidation) [31] and phase II BaP metabolites involving conjugation reactions (e.g., transferase). The primary metabolism—encompassing essential endogenous metabolites such as amino acids, sugars, and nucleotides—even if its content is influenced by BaP exposure [30], is expected to remain unchanged in terms of mass-to-charge ratio (*m*/*z*) under the experimental conditions between RHE exposed to BaP versus the ones exposed to BaP-d12. In contrast, differences are anticipated in the *m*/*z* values corresponding to compounds directly generated by BaP exposure, namely the xenometabolites. Mass spectrometric analysis is therefore expected to reveal distinct isotopically labeled signals, enabling the identification and characterization of the specific metabolic footprint associated with BaP exposure in RHE.

The obtained MS spectra of extracts from BaP-treated epidermis were compared to those from BaP-d12-treated epidermis. Matches were identified where applicable by determining the deuterium–hydrogen exchange number (Δn(^2^H–H)).

The Δn(^2^H–H) corresponds to the number of deuterium-to-hydrogen exchanges between a labeled molecule and its non-labeled counterpart. Thus, between BaP and BaP-d12, the Δn(^2^H–H) is set at 12 maximum as BaP contains 12 H atoms when BaP-d12 has 12 ^2^H instead. Consequently, the Δn(^2^H–H) between a labeled metabolite derived from BaP-d12 and the same non-labeled metabolite derived from BaP will be equal to or less than 12. This difference results in a mass shift (Δ*m*/*z*) observable by HRMS, defined and calculated according to the following formula:

Δ*m*/*z* [(BaP − X) − (BaP-d12 − X)] = Δn(^2^H–H) × Δ*m*/*z* (^2^H–H), (1)

where X is the biotransformation item (functionalization and/or conjugation); BaP-X and BaP-d12-X are the BaP and BaP-d12 xenometabolites, respectively, with 0 < Δn(^2^H–H) ≤ 12 and Δ*m*/*z* (^2^H–H) = 1.00628

Consequently, features (*m*/*z* associated with a retention time, RT) that show Δ*m*/*z* values between the BaP and BaP-d12 experiments in accordance with the formula above highlight the presence of deuterium labeling and thus a direct origin from BaP.

### 3.2. Search for Xenometabolites

To assess the utility of stable isotope labeling (SIL) and identify potential xenobiotic metabolites, we analyzed LC-HRMS datasets from mock-treated (1.25‰ DMSO, n = 1), BaP-treated (n = 3) and BaP-d12-treated samples (n = 3). To enable the identification of BaP-derived xenometabolites through comparative analysis samples, BaP-treated tissues were labeled as “sample,” while BaP-d12-treated tissues were designated as “labeled.” The mock-treated tissue, labeled as “blank”, consisted of RHE exposed to the vehicle only, and served as a biological blank to differentiate treatment-specific features from endogenous metabolites. The peak alignment and detection for each of the three datasets were carried out separately using Compound Discoverer 3.1, applying optimized automatic filtering criteria.

After the first analysis including peak picking and alignment between all the samples, a total of 6400 features were automatically extracted from one or more samples. However, not all of these correspond to BaP metabolites. This result indicates that numerous endogenous low-molecular-weight metabolites were detected in skin exposed to either DMSO, BaP or BaP-d12. To focus on features of interest, a series of filters was applied to eliminate those unlikely to represent BaP metabolites. First, features labeled as “background is false” were retained—meaning any signal present in both BaP and BaP-d12 samples but also detected in the vehicle-treated control was excluded. Second, only features with a retention time (RT) shorter than 2.3 min were considered, as BaP metabolites are expected to be more polar than the parent compound and therefore elute earlier in reverse-phase chromatography. Finally, a molecular mass threshold of 252.0939 Da—corresponding to the exact mass of native BaP—was applied to retain only those features consistent with metabolite formation, based on the assumption that biotransformation introduces at least one additional functional group.

**Figure 3:**
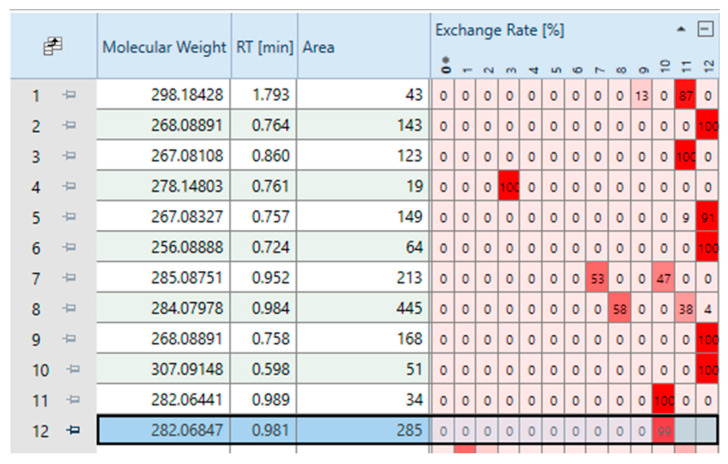
Section of the table displayed in Compound Discoverer^®^ after applying the first filter, showing for each variable the exact mass of the neutral molecule, retention time, associated peak area, and exchange rate (in %) for each possible deuterium–hydrogen exchange number (ranging from 0 to 12).

Following the application of these filters, 227 features remained. A subset of these features, as visualized in Compound Discoverer^®^, is presented in Figure 3.

It is important to note that certain PAHs preferentially ionize as radical cations ([M]^+^•). However, the signal extraction parameters used in the data processing pipeline do not natively support the direct assignment of such radical species. To address this limitation, a mathematical workaround was implemented to enable the automated identification of these ionization forms.

To further eliminate false positive peaks, paired-peak filtering was applied. This technique identifies LC/HRMS peak pairs generated from both unlabeled and labeled BaP. This second filter retained only features that met the following criteria: (i) a deuterium–hydrogen exchange, i.e., features with a Δn(^2^H–H) greater than 0, and (ii) a molecular formula containing 20 carbon atoms. However, it is important to note that the retention times (RTs) of the ^2^H-labeled compounds on the reverse-phase LC column were shorter than those of their unlabeled counterparts. This difference can be attributed to the shorter hydrogen bonding distance of ^2^H compared to ^1^H, as well as the position and number of ^2^H labels [32]. Therefore, to account for this deviation, a ΔRT threshold of 0.2 min was applied. 

**Figure 4:**
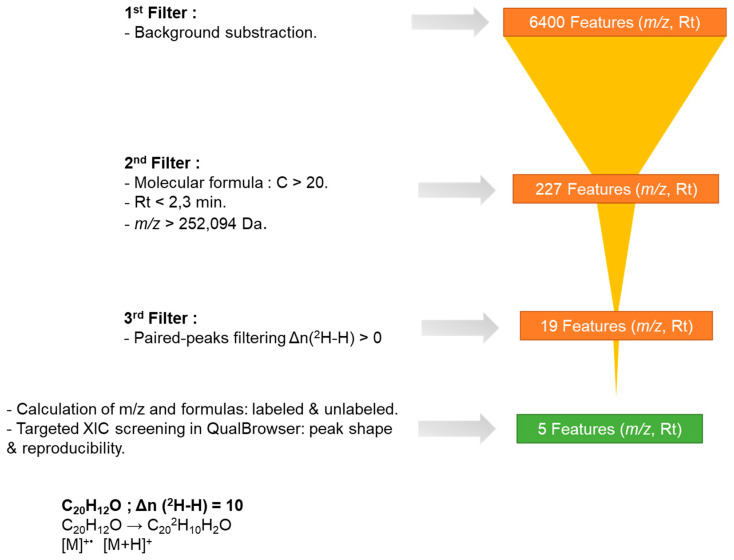
A tiered peak filtering approach to prioritize candidates of BaP metabolites.

Of the 227 features previously identified, 19 met the criteria of the second filtering step (Figure 4). However, the presence of false positives could not be entirely excluded as peak picking algorithms are known to generate false peaks [33]. To ensure the analytical relevance of these features, each was manually checked using the QualBrowser module of Xcalibur. This verification included checks of peak shape, signal quality, and alignment between the labeled and unlabeled forms. Features were only retained if the target molecule was detected in at least two out of three biological replicates—a criterion applied to account for biological variability and sensitivity. Additionally, the signal-to-noise ratio (S/N) between the labeled and non-labeled samples, as well as the biological blank, had to exceed a value of 3 to be considered analytically valid.

To this end, based on the neutral molecule M extracted by the software and its associated Δn(^2^H–H), we calculated the exact masses corresponding to the [M]^+^• and [M+H]^+^ ions of both the non-labeled and labeled molecules. For example, from the neutral mass M = 268.08882, the deduced molecular formula is C_20_H_12_O. This molecule may be detectable as [M]^+^• and [M+H]^+^ at *m*/*z* 268.08827 and *m*/*z* 269.09609, respectively. If the determined exchange number is 10, the labeled molecular formula would be C_20_^2^H_10_H_2_O (with 10 deuterium atoms), and the corresponding ions would be detected at *m*/*z* 278.15103 ([M]^+^•) and *m*/*z* 279.15885 ([M+H]^+^), respectively. It is worth noting that some features display multiple exchange numbers, as shown in Figure 3. This is likely due to in-source exchange during LC-HRMS analysis. In such cases, all labeled molecular formulas were considered.

As a result, five features ultimately passed this final filter and can be considered as BaP metabolites. These are described in Table 1.

The five features correspond to hydroxylated, dihydroxylated, and quinone derivatives. These characterizations (level3) were made possible by HRMS (with a mass tolerance of ±10 ppm) which allows molecular formula assignment following the recommendations of the Chemical Analysis Working Group [34]. These derivatives have also been reported in the literature [35]. As described in the literature, hydroxylated and dihydroxyl derivatives can also be detected as [M+H–H_2_O]^+^ ions, which complicates molecular formula assignment (features 1, 2, and 5) [35].

It is important to note that BaP itself appears in this list and is considered a positive control for the data processing workflow.

## 4. Discussion

Understanding BaP skin metabolism is key for achieving better skin health. In this study, we chose to investigate the BaP xenometabolism using in vitro tests. Despite their inherent inability to fully replicate in vivo complexities—such as complete metabolic and clearance capacities or intricate enzyme regulation—isolated in vitro skin models are crucial for systematic screening studies, providing fundamental insights into mechanisms of action [36].

Even if the primary route of contact with the skin is indeed topical, we opted for a basal treatment mimicking systemic exposure via the vehicle media to investigate the direct cellular effects of BaP once it is bioavailable to the living skin cells. We ensured a more homogeneous exposure to cells, allowing us to focus on the cellular response itself. Our basal treatment therefore simulates a scenario where xenometabolites, having entered the body via inhalation or other routes, are then delivered via blood circulation [37].

The identification of xenometabolites derived from exogenous chemicals in complex biological matrices remains a key challenge in untargeted analysis. High-resolution mass spectrometry (HRMS) offers the sensitivity and resolution needed to detect a wide range of TPs, but the structural elucidation and discrimination of xenobiotic-derived features from the endogenous background require advanced data processing strategies. Among the most widely used are chemometric analyses, mass defect-based filtering, in silico metabolism prediction tools, and stable isotope labeling—each with specific advantages and limitations.

Mass defect analysis, including Kendrick mass defect (KMD) and related techniques, facilitates the detection of structurally related metabolite series by grouping ions with predictable elemental compositions or transformation patterns [38]. This approach is particularly useful for identifying homologous metabolites generated through oxidative metabolism, conjugation (e.g., glucuronidation or sulfation), or other phase I/II reactions. Despite its usefulness, mass defect analysis may misclassify unrelated compounds with coincidentally similar mass defects and generally requires complementary information such as MS/MS data for confident annotation. Additionally, this approach may fail to account for cases where parent compounds undergo extensive cleavage into smaller TPs whose mass defects diverge significantly from those of their precursors. This scenario might occur, for example, when benzo[a]pyrene (BaP) is degraded by bacteria [39,40].

Chemometric techniques, including principal component analysis (PCA), partial least squares discriminant analysis (PLS-DA), and clustering algorithms, are commonly employed to extract statistically significant features from LC-HRMS datasets [41]. These multivariate tools assist in distinguishing treated from untreated conditions in an exhaustive manner and can help reveal potential xenometabolites. However, these models are data-driven and unsupervised, which may lead to false positives if not rigorously validated. Overfitting, batch effects, and collinearity remain critical pitfalls, especially in studies with limited replicates. Major changes in the dimensionality and complexity of datasets lead to a significant shift in knowledge discovery [42]. The complexity of metabolomics also presents great challenges for chemometrics to deal with such massive high-dimensional data [43]. These approaches, if used wisely, could help identify a series of endogenous metabolites (such as ATP) that have been modified due to BaP exposure, alongside the xenometabolites. Therefore, a second filtering step would be helpful to distinguish endogenous from xenobiotic metabolisms.

In silico metabolite prediction tools, such as Meteor Nexus (Lhasa Ltd.), BioTransformer, or the ADMET Predictor, offer structure-based simulation of likely metabolic transformations [44]. These tools can guide experimental design, prioritize candidate metabolites, and aid annotation by predicting possible mass-to-charge (*m*/*z*) values and molecular formulas. Despite their growing utility, their predictive power is constrained by database coverage and the inherent assumptions of their transformation rules, limiting their accuracy for novel chemical entities or unusual metabolic reactions [45].

Stable isotope labeling (SIL) offers a distinct advantage in excluding interference from endogenous metabolites resulting from xenobiotic exposure, especially in biological samples, as these endogenous compounds lack isotopic labels and therefore do not generate the characteristic ion pairs [21]. Since a labeled compound possesses physicochemical properties nearly identical to those of its unlabeled counterpart, both undergo the same transformation processes, resulting in ion pairs of labeled and unlabeled TPs with a consistent characteristic mass difference. These pairs exhibit similar chromatographic retention and equivalent mass spectrometric ionization efficiencies, and are expected to exhibit similar biological effects, as enzymes process both similarly. Furthermore, the ratio of the labeled to unlabeled ion pairs can be employed as an additional filter to eliminate interfering ions. This approach offers a comprehensive way to decipher BaP metabolism. In this study, the LC-HRMS analysis combined with isotopic labeling allowed the unexpected identification of five phase I metabolites present in epidermal extracts.

However, this methodology presents limitations that complicate data interpretation. In reverse-phase LC, the RTs of ^2^H-labeled compounds elute slightly faster than those of the unlabeled form [46]. This difference in elution between the labeled and unlabeled compounds should be explicitly accounted for during data analysis. Manual checking for the last 19 candidates features helps us validate our assignments. Furthermore, deuterium-labeled molecules, as opposed to their carbon-13-labeled counterparts, can be less stable during LC-HRMS analysis due to potential exchange with the mobile phase. As a result, a single metabolite may appear with multiple deuterium exchange numbers, leading to redundancy in the detected features; this can be tackled by choosing alternative labeling when available.

Moreover, a key limitation of this study was the use of real-life concentrations which means low working concentrations, which, together with substantial losses due to adsorption and the relatively lower sensitivity of LC-HRMS, necessitated lowering the ion detection threshold. This initially resulted in a large number of detected features. However, the application of intelligent peak filtering effectively reduced this number, enabling a focused analysis of the most probable BaP metabolites.

When exposing the SkinEthic model to BaP via the systemic route, we demonstrated that the model exhibits metabolic capabilities. Through the use of SIL, HRMS, and a data-mining strategy, we successfully confirmed five BaP metabolites, most of which have previously been reported in lung cells [18]. BaP-diones are produced through enzymatic oxidation mediated by cytochrome P450 peroxidase or monooxygenase pathways. BaP-OH is formed directly from BaP via CYP1A1/1A1B activity, whereas BaP-diones can also be derived from the further metabolism of BaP-diols [18]. The BaP-diols, which likely originate from epoxide intermediates, were not detectable by MS, possibly due to their instability. The enzyme system responsible for these metabolic conversions is also expressed in human skin and a reconstructed human epidermis (RHE) model [36]. These BaP metabolites are known to induce reactive oxygen species (ROS) and singlet oxygen production, which can be effectively neutralized by endogenous antioxidants such as superoxide dismutase (SOD) and glutathione [37]. Broader omics analyses, i.e., transcriptomics or proteomics, should confirm the metabolic pathways proposed. It is important to note that all experiments were conducted using a single tissue lot, which represents a limitation for the generalizability of the results. Future studies could explore inter-lot variability.

This discovery of BaP metabolites opens new pathways for protecting against pollution. For example, it can guide the development and use of antioxidants to mitigate the harmful effects caused by exposure to such pollutants. By understanding how BaP is metabolized, we can better design strategies to neutralize its toxicity and reduce environmental and health risks.

Future work on SIL could take inspiration from the IsoNet strategy developed by Gao et al., which presents a transformative, hypothesis-free approach for uncovering unknown metabolic reactions [47]. This method demonstrates how SIL can go beyond filtering xenometabolites to systematically map biotransformations, thereby deepening our understanding of both endogenous and xenobiotic metabolism [47].

## 5. Conclusions

This study demonstrated the feasibility and advantages of combining HRMS with deuterium isotopic labeling to investigate the metabolism of benzo[a]pyrene (BaP) in skin cells. The use of deuterium-labeled BaP allowed selective tracing of metabolite formation by identifying characteristic mass shifts corresponding to the sole deuterium–hydrogen exchanges, thereby overcoming matrix interferences common in complex biological samples. By analyzing ion pairs with distinct isotope patterns and abundance ratios, false positives from the matrix were effectively excluded, facilitating the comprehensive identification of possible BaP metabolites. Using this approach, several phase I metabolites—including hydroxylated, dihydroxylated, and quinone derivatives—were identified in epidermal extracts. This methodology represents a powerful tool for profiling xenobiotic metabolism in complex biological matrices at trace levels, especially when conventional non-labeled approaches are insufficient.

## Figures and Tables

**Table 1:** List of BaP metabolites detected through isotopic labeling. BaP is included as a positive control of the workflow.

Feature	Molecular Mass (M)	Molecular Formula	RT (min)	Modification	Identification
–	252.09390	C_20_H_12_O	2.2	–	BaP
1	268.08882	C_20_H_12_O	1.5	+OH	Hydroxylated derivative
2	268.08882	C_20_H_12_O	0.8	+OH	Hydroxylated derivative
3	282.06808	C_20_H_10_O_2_	1.7	+2O	Quinone derivative
4	282.06808	C_20_H_10_O_2_	1.0	+2O	Quinone derivative
5	284.08372	C_20_H_12_O_2_	0.9	+2OH	Dihydroxylated derivative

## Data Availability

The original contributions presented in this study are included in the article material. For further inquiries, please contact the corresponding author(s).

## Abbreviations

The following abbreviations are used in this manuscript:

ACN	Acetonitrile
APCI	Atmospheric Pressure Chemical Ionization
ATP	Adenosine Triphosphate
BaP	Benzo[a]pyrene
CYP	Cytochrome P450 Monooxygenases
DMSO	Dimethyl Sulfoxide
FWHM	Full Width at Half Maximum
HRMS	High-Resolution Mass Spectrometry
KMD	Kendrick Mass Defect
LC-HRMS	Liquid Chromatography–High-Resolution Mass Spectrometry
*m*/*z*	Mass-to-Charge Ratio
mEH	Microsomal Epoxide Hydrolase
MS	Mass Spectrometry
MS/MS	Tandem Mass Spectrometry
PCA	Principal Component Analysis
PAH	Polycyclic Aromatic Hydrocarbon
PDA	Photodiode Array
PLS-DA	Partial Least Squares Discriminant Analysis
ppm	Parts Per Million
QTOF-MS	Quadrupole Time-of-Flight Mass Spectrometry
RHE	Reconstructed Human Epidermis
RT	Retention Time
SIL	Stable Isotope Labeling
S/N	Signal-to-Noise Ratio
SOD	Superoxide Dismutase
TPs	Transformation Product
UFP	Ultrafine Particle
UHPLC	Ultra-High-Performance Liquid Chromatography
UV	Ultraviolet
UVA1	Ultraviolet A1 (350–400 nm)
